# Epigenetic heterogeneity in cancer

**DOI:** 10.1186/s40364-019-0174-y

**Published:** 2019-10-31

**Authors:** Mingzhou Guo, Yaojun Peng, Aiai Gao, Chen Du, James G. Herman

**Affiliations:** 1 Department of Gastroenterology & Hepatology, Chinese PLA General Hospital, #28 Fuxing Road, Beijing, 100853 China; 2 State Key Laboratory of Esophageal Cancer Prevention and Treatment, 40 Daxue Road, Zhengzhou, Henan 450052 China; 3 The Hillman Cancer Center, University of Pittsburgh Cancer Institute, 5117 Centre Ave., Pittsburgh, PA 15213 USA

**Keywords:** Epigenetics, Intratumor heterogeneity, Epigenetic intratumor heterogeneity, Epigenetic machinery, Microenvironment

## Abstract

Phenotypic and functional heterogeneity is one of the hallmarks of human cancers. Tumor genotype variations among tumors within different patients are known as interpatient heterogeneity, and variability among multiple tumors of the same type arising in the same patient is referred to as intra-patient heterogeneity. Subpopulations of cancer cells with distinct phenotypic and molecular features within a tumor are called intratumor heterogeneity (ITH). Since Nowell proposed the clonal evolution of tumor cell populations in 1976, tumor heterogeneity, especially ITH, was actively studied. Research has focused on the genetic basis of cancer, particularly mutational activation of oncogenes or inactivation of tumor-suppressor genes (TSGs). The phenomenon of ITH is commonly explained by Darwinian-like clonal evolution of a single tumor. Despite the monoclonal origin of most cancers, new clones arise during tumor progression due to the continuous acquisition of mutations. It is clear that disruption of the "epigenetic machinery" plays an important role in cancer development. Aberrant epigenetic changes occur more frequently than gene mutations in human cancers. The epigenome is at the intersection of the environment and genome. Epigenetic dysregulation occurs in the earliest stage of cancer. The current trend of epigenetic therapy is to use epigenetic drugs to reverse and/or delay future resistance to cancer therapies. A majority of cancer therapies fail to achieve durable responses, which is often attributed to ITH. Epigenetic therapy may reverse drug resistance in heterogeneous cancer. Complete understanding of genetic and epigenetic heterogeneity may assist in designing combinations of targeted therapies based on molecular information extracted from individual tumors.

## Background

Cellular heterogeneity is a well-recognized attribute of both normal and neoplastic tissue [1]. Tumor morphologic heterogeneity has long been recognized by pathologists and forms the basis of many tumor grading prognostic classification systems [2]. Within a tumor, there is diversity in tumor cell proliferation, immune infiltration, differentiation status, and necrosis that can differ between microscopy fields [2]. In healthy tissue, the stroma functions are the main barrier against tumorigenesis; however, the presence of transformed tumor cells initiates crucial changes that can convert this environment into one that supports cancer progression [3]. Regional differences in extracellular microenvironment such as hypoxia, acidity and the presence of growth factors exist within a tumor and actively shape its development [3]. Normal fibroblasts typically suppress tumor formation, while cancer-associated fibroblast (CAFs) can significantly promote tumorigenesis [4–6]. Compared to normal tissue fibroblasts, CAFs have increased proliferation, enhanced extracellular matrix production and unique cytokine secretion [7]. Other mesenchyme-derived cell types, such as adipocytes, vascular endothelial cells and immune cells, as well as extracellular matrix, can also contribute to tumor growth and progression [8]. These stromal components may be different in many tumors.

By analyzing normal esophageal mucosa, esophageal dysplasia and esophageal squamous cell carcinoma, our previous study found that accumulation of aberrant tumor suppressor gene promoter region methylation is similar to classic gene mutation accumulation that occurs during tumor progression [9, 10]. In 1953, Slaughter et al. proposed the concept of field cancerization (also known as field defect) to explain the occurrence of multiple primary tumors, local recurrence, abnormal tissue surrounding the cancer and multifocal areas of precancerous change [11]. Phenotypic and functional heterogeneity are hallmarks of human cancers [12]. Tumor genotype variations among tumors within different patients are known as interpatient heterogeneity [13], and variability among multiple tumors of the same type arising in the same patient is referred to as intra-patient heterogeneity [13]. Subpopulations of cancer cells with distinct phenotypic and molecular features within a tumor is called intratumor heterogeneity (ITH) [13]. ITH is characterized by substantial phenotypic cell-to-cell variability, including differences in motility, metabolism, angiogenesis, proliferation, immunogenesis, and metastatic potential [14, 15]. ITH also includes heterogeneity of the tumor microenvironment [16, 17].

Phenotype heterogeneity of cells within tumors was noted in the earliest days of cancer biology [18]. Since the discovery that formation of tumors is dependent on the acquisition of oncogenic mutations, the existence of heterogeneity in clinically important traits was attributed to genetic diversity. Current approaches for molecular biomarker testing and targeting therapy are mainly focused on interpatient tumor heterogeneity [13]. However, there is growing recognition that ITH within the same patient is clinically relevant because the status of predictive biomarkers used for making clinical decisions may evolve during tumor progression, in particular for metastatic dissemination of the primary tumor to a distant organ or for established metastatic disease under the selection pressure of treatment [13].

The phenomenon of ITH is commonly explained by Darwinian-like clonal evolution of a single tumor [18]. Despite the monoclonal origin of most cancers, new clones arise during tumor progression due to the continuous acquisition of mutations. This promotes division into subclones and causes an increase in ITH [19]. Mutations that occur early in tumor evolution are present in all regions and almost all tumor cells harbor them. While, mutations that occur later or in the latest tumor progression are present in only some regions or only one subclone. These later occurring mutations are the basis for genetic ITH [2]. Heterogeneity in this field results in differences in features of subclones within a tumor, including different proliferation rates and different responses to treatment. However, the dominance of gene-centric views has been challenged with the rapid development of research within the cancer stem cell hypothesis, thus bringing non-genetic sources of phenotypic variability into focus [20]. In this review, we discuss the contributions of epigenetics to tumor phenotypic heterogeneity, mainly focused on the disruption of “epigenetic machinery”.

## Genetic heterogeneity in cancer

Historically, research has focused on the genetic basis of cancer, particularly mutational activation of oncogenes or inactivation of tumor-suppressor genes (TSGs). Since Nowell proposed the clonal evolution of tumor cell populations in 1976, tumor heterogeneity, especially ITH, was actively studied [18]. However, many biological aspects of tumor heterogeneity remain unknown [21]. The analysis of multiple biopsies from the same tumor can reveal the spatial composition and evolutionary trajectory of subclones. The clonal and subclonal composition of each tumor can be used to construct distance-based phylogenetic trees. Mutations present in all samples of a tumor are inferred to be acquired by early precursor cells that clonally expanded (clonal mutations), represented by truncal events on the evolutionary tree, and mutations present in only a subset of samples are inferred to be later events, acquired at some point during or after the initial clonal expansion (subclonal mutations) [22, 23].

Gerlinger and colleagues obtained tumor samples from four patients with renal-cell cancer before and after treatment and took multiple samples from each parent’s primary and metastatic tumor sites. Analysis revealed that 63 to 69% of mutations in single biopsies were not detectable across every tumor region of the same patient [24]. Thus, a single tumor biopsy, the standard of tumor diagnosis and the cornerstone of personalized-medicine decisions, cannot be considered representative of the landscape of genomic abnormalities in a tumor. ITH is found in most, probably all, solid human tumors. Underestimation of tumor heterogeneity may lead to a serious flaw in cancer diagnosis and treatment selection.

## Disruption of “epigenetic machinery” in cancer

In the nucleus of eukaryotic cells, chromatin provides the scaffold for the packaging of the entire genome. The basic functional unit of chromatin is the nucleosome, and it contains 147 base pairs of DNA wrapped around a histone octamer, with two copies each of histones H2A, H2B, H3 and H4. The epigenome consists of specific covalent modifications of chromatin components, including DNA methylation and histone modifications. These covalent modifications control the structure and function of chromatin. Epigenetic regulation of gene expression is mainly dependent on DNA methylation and histone modifications, without intrinsic changes in the DNA sequence, and epigenetic change is heritable [25]. Noncoding RNA, ubiquitylation and sumoylation are also included in the field of epigenetics [26]. The regulators of “epigenetic machinery” are divided into “writers” (enzymes that establish DNA methylation or histone modifications), “erasers” (proteins that remove these marks) and “readers” (proteins that bind to modifications and facilitate epigenetic effects). Protein complexes that position the nucleosomes across the genome are called “movers” [26].

In mammals, DNA methylation occurs predominantly at the 5′ position of cytosine forming cytosine guanine dinucleotides. This modification is carried out by DNA methyltransferases (DNMTs), enzymes that use S-adenosymethionine (SAM) as a methyl group donor. DNA methylation patterns are established and maintained by three DNMTs: DNMT1, DNMT3A and DNMT3B. Depending on the genomic location, DNA methylation may have different biological functions. Methylation in gene promoter regions is typically associated with gene repression, while methylation in the gene body is usually associated with active gene expression. Increasing evidence has shown that intergenic regions contain many regulatory elements, such as enhancers, silencers and non-coding RNAs, and their function may also be affected by DNA methylation. Early epigenetic research typically focused on gene promoter regions [26].

For a long time, 5-methylcyctosine (5mC) was considered to be a relatively permanent mark, but this view changed abruptly with the discovery of the function of the ten-eleven translocation (TET) proteins, TET1, TET2 and TET3. The TET gene family was initially identified as a result of a chromosomal rearrangement (t(10;11)), (q22;q23) involving TET1 and MLL, which encodes one of the histone H3 lysine 4 (H3K4) methyltransferases in acute myeloid and lymphocytic leukemias [27]. The TET family utilizes two key co-factors, Fe (II) and 2-oxoglutatate (2-OG), to successively oxidize the methyl group of 5mC to hydromethyl, forml or carboxyl groups, thus forming 5-hydroxymethylcytosine (5hmC), 5-formylcytosine (5fC) and 5-carboxylcytosine (5caC), together termed ‘oxi-mC [28]. 5-hmC can actively facilitate DNA demethylation by inhibiting UHRF1/DNMT1 complex binding to DNA for methylation maintenance. 5fC and 5caC can be excised by the DNA repair enzyme thymine-DNA glycosylase (TDG), followed by replacement with unmodified cytosine through a base pair mechanism [29]. TET proteins bind preferentially to unmethylated CpGs within CpG-rich genomic regions (termed CpG islands), thus maintaining CpG islands (CGIs) in a hypomethylated state, and 5hmC is associated with active transcription [30]. Downregulation of TET proteins and loss of 5hmC are viewed as new epigenetic hallmarks of human cancer [31]. Isocitrate dehydrogenases (IDH) are key metabolic enzymes that function in the tricarboxylic acid (TCA) cycle; they convert isocitrate to 2OG using NADP+/NADPH as factors. 2OG is an essential cofactor for dioxygenases including TET proteins and the JmjC family of lysine demethylases. Among the three IDH enzymes, IDH1 and IDH2 are frequently mutated in glioma and hematological malignancies [32].

Histones are modified by different enzymes, including histone acetyltransferases (HATs), histone deacetylases (HDACs), histone methytransferases (HMTs) and histone demethylases (HDMs). HDACs are enzymes responsible for removing the acetyl group from lysine residues in histones [26, 33]. There are various types of histone tail modifications, such as acetylation, methylation, ubiquitination, among others. These modifications regulate gene expression through their interactions with chromatin-associated proteins in marking regions of transcriptionally active euchromatin and inactive heterochromatin, inducing transcriptional activation or repression. For example, in the promoter region, acetylated histone H3, and di- or tri-methylated histone H3 lysine 4 (H3K4me2, H3K4me3) represent activation of gene expression. Repressed promoters are usually marked with tri-methylated histone H3 lysine 27 (H3K27me3) and tri-methylated histone H3 lysine 9 (H3K9me3), which correlate with constitutive heterochromatin and DNA methylation [26].

**Fig. 1:**
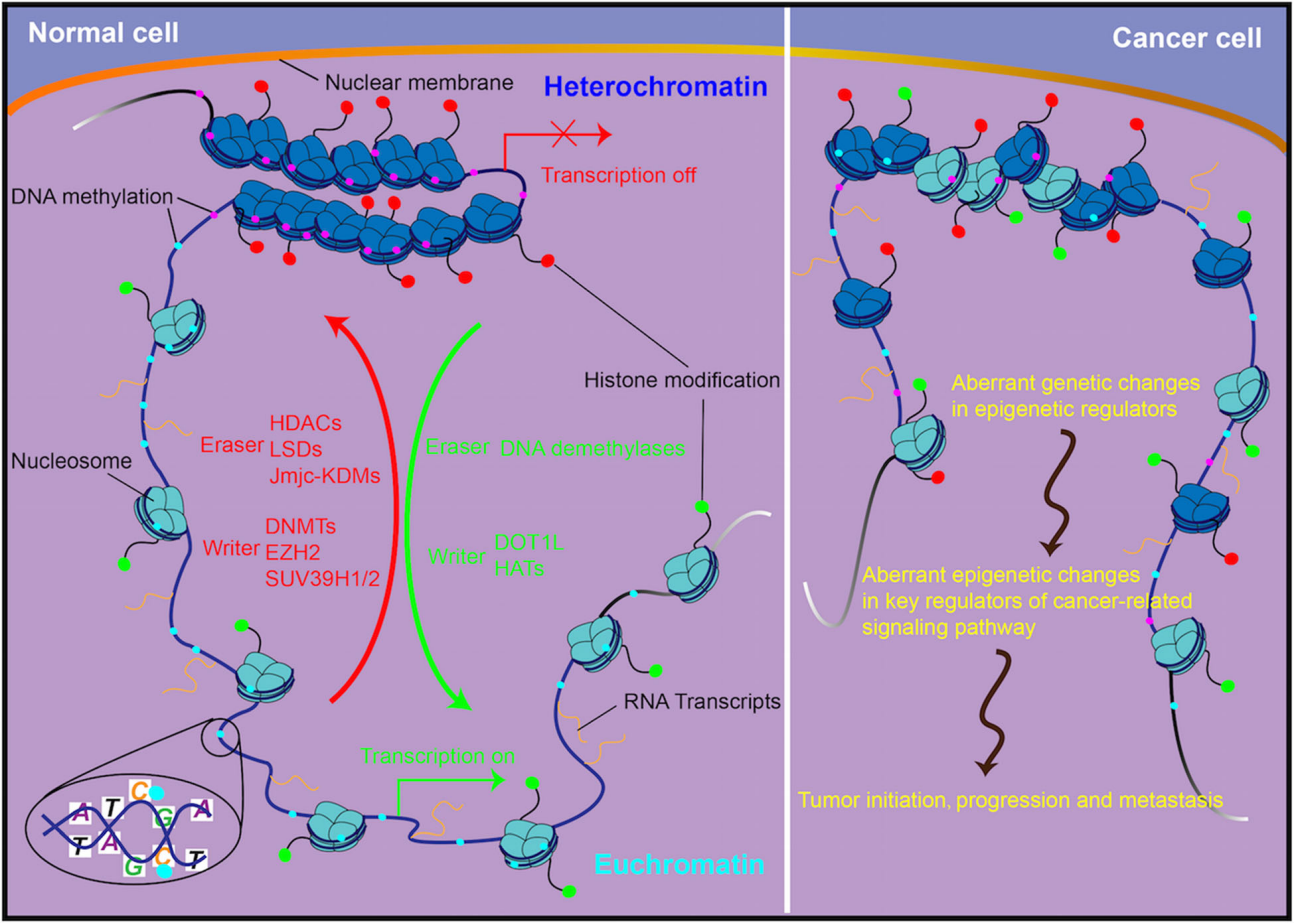
Disruption of the “epigenetic machinery” in cancer. Aberrant changes of major players of “epigenetic machinery” during cancer initiation, progression and metastasis. HATs, DOT1L, DNMT, EZH2, SUV39H1/2: representative writers (enzymes that establish DNA methylation or histone modifications); HDACs, JmjC–KDMs, LSDs, DNA demethylase: representative erasers (proteins that remove DNA methylation or histone modification marks)

It is clear that disruption of the "epigenetic machinery" plays an important role in cancer development (Fig. 1). The recognition of an epigenetic component in tumorigenesis, or the existence of a cancer ‘epigenome’, has led to new opportunities for the understanding, detection, treatment, and prevention of cancer [33, 34]. DNA methylation is the most frequently found abnormal epigenetic change in human cancers. Global genomic DNA hypomethylation and promoter region hypermethylation have been extensively studied in human cancer [35, 36]. Aberrant epigenetic changes occur more frequently than gene mutations in human cancers. For example, epigenetic silencing of CDK2NA and MLH1 is much more common than mutational inactivation of either of these two well-recognized driver genes [26]. Beyond lifestyle determinants, the role of environmental factors as determinants of DNA methylation has gained considerable attention [35]. The epigenome is at the intersection of the environment and genome [37]. Epigenetic dysregulation occurs in the earliest stage of cancer. For example, DNA methylation was shown to be altered in the normal tissue of lung cancer patients [38]. A second example is that tumor suppressor genes were methylated in the early stage of esophageal squamous cell carcinogenesis, and accumulation of promoter region methylation was correlated with cancer progression [9, 39]. In addition, recently discovered mutations in the epigenetic apparatus likely contribute to epigenetic disruption in cancer [37]. DNA methylation is the most useful epigenetic marker for human disease studies because it is stable over a period of decades and is present in archival specimens, including paraffin blocks [40]. Aberrant DNA methylation is involved in the major components of cell cycle, DNA damage repair, Wnt, TGF-β, NF-kB and other cancer-related signaling pathways [41–43]. Additional information is provided in Table 1.

**Table 1:** Aberrant epigenetic changes of the major components in cancer-related signaling pathways

Signaling pathway	Gene	Major role	Alteration	Tumor type	Ref
Cell cycle regulation	p16 (CDKN2A)	G1-S transition	promoter hypermethylation	oral and oropharyngeal squamous cell carcinoma, HCC, GC, CRC, esophageal, lung and ovarian cancer	[44, 45]
	CHFR	G2-M transition	promoter hypermethylation	NSCLC, GC, CRC, BC,, HCC, nasopharyngeal, bladder, esophageal, cervical and endometrial cancers	[46]
	RASSF1A	M-phase progression	promoter hypermethylation	BC, head and neck cancer, gynecological, lung, prostate, bladder, brain, gastrointestinal, renal and renal cancers, sarcoma melanoma	[47]
	Chk2	checkpoint kinase 2, damage sensors	promoter hypermethylation	Glioma, Hodgkin’s lymphoma, NSCLC	[48–50]
	ATM	cell cycle checkpoint kinase	promoter hypermethylation	BC, gastric lymphoma, glioma, colonic cancer, adenoma	[51]
DNA damage repair	BRCA1	HR repair	promoter hypermethylation	NSCLC, CRC, breast, ovarian, bladder and pancreatic cancers	[52–55]
	BRCA2	HR repair	promoter hypermethylation	NSCLC, BC and ovarian cancers	[56]
	FANCF	FA complementation group F	promoter hypermethylation	head and neck cancers, NSCLC, ovarian, breast and cervical cancer	[57]
	XRCC5	X-ray repair cross complementing 5, component of NHEJ	promoter hypermethylation	NSCLC, glioma	[56, 58]
	WRN	Werner syndrome RecQ like helicase, component of BER	promoter hypermethylation	NSCLC, CRC, GC, prostate, breast and thyroid cancers, non-Hodgkin lymphoma, chondrosarcomas	[59]
	MSH2	MutS family protein, MMR ATPase	promoter hypermethylation	hereditary nonpolyposis colorectal cancer, HCC	[60, 61]
	RAD23B (hHR23B)	RAD23 homolog B, NER protein	promoter hypermethylation	myeloma	[62]
Wnt pathway	WNT5A	ligand	promoter hypermethylation, histone modification	CRC	[63]
	WNT2	ligand	histone modification	CRC	[64]
	WIF1	sequesters Wnt proteins	promoter hypermethylation	HCC, GC, BC, CRC, lung cancer	[65–68]
	DKK	LRP5/6 antagonist	promoter hypermethylation, histone modification	NSCLC, BC, CRC, GC, HCC	[63, 69–71]
	Cadherin	CTNNB1 translocation	promoter hypermethylation	BC, NSCLC, CRC, GC, HCC, ESSC, CLL, pancreatic, bladder and prostate cancers	[72]
	APC	binds CTNNB1 to destruction complex	promoter hypermethylation, histone modification	GC, CRC, ESCC, BC, NSCLC, HCC and prostate cancer	[73]
	GSK3β	phosphorylates CTNNB1	promoter hypermethylation	CRC	[74]
TGF-β pathway	RUNX3	interacts with SMADs	promoter hypermethylation	GC, HCC, CRC, BC, lung, bladder and pancreatic cancers	[75]
	SMAD6/7	inhibits TGF-β-induced SMAD3 activation	promoter hypermethylation, histone methylation	Lung cancer	[76]
	DACH1	interacts with NCoR and SMAD4	promoter hypermethylation	GC, ESSC, CRC, HCC, endometrial, lung and renal cancers	[42, 77–79]
	FBXO32	target of TGF-β signaling pathway	promoter hypermethylation	ESSC, GC and ovarian cancer	[80, 81]
NF-κB pathway	p65 (RelA)	major functional subunit of NF-κB	histone modification	CRC, osteosarcoma	[82, 83]
	ZNF382	inhibitor of NF-κB	promoter hypermethylation	GC, ESCC, CRC, BC and nasopharyngeal cancer	[84]
	ZNF545	inhibitor of NF-κB	promoter hypermethylation	HCC	[43]
	CXCL14	inhibitor of NF-κB	promoter hypermethylation	CRC	[85]
Akt pathway	ADAMTS9	inhibitor of Akt signaling	promoter hypermethylation	CRC	[86]
	RAI2	inhibitor of Akt signaling	promoter hypermethylation	CRC	[87]
	HIN-1	inhibitor of Akt signaling	promoter hypermethylation	NSCLC	[88]
p53 pathway	RASSF10	agonist of p53 signaling	promoter hypermethylation	CRC, HCC, papillary thyroid carcinoma	[89, 90]
	BCL6B	agonist of p53 signaling	promoter hypermethylation	HCC, CRC, GC	[91, 92]
	DLEC1	target of p53 signaling	promoter hypermethylation	ESCC, nasopharyngeal, lung carcinomas	[93]
Retinoid acid pathway	CRBP1	retinol-binding protein	promoter hypermethylation	CRC, ESSC, gastrointestinal carcinoma, lymphoma, prostate cancer	[94–98]
	RARbeta2	retinoic acid receptor	promoter hypermethylation	BC, lymphoma, gastrointestinal carcinomas, bladder cancer	[97, 99, 100]
Hedgehog pathway	PTCH1	primary receptor	promoter hypermethylation, histone modification	GC, BC, medulloblastoma, basal cell carcinomas	[101–103]
	SHH	ligand	promoter hypomethylation, histone modification	GC, BC	[104, 105]
	HHIP	ligand sequestering	promoter hypermethylation	LAC, GC, HCC, medulloblastoma, pancreatic cancer	[106]
	GLI1	transcription activator	histone modification	BC	[107]
	GLI3	transcription repressor	promoter hypomethylation	GC	[108]

## The interplay between genetics and epigenetics

As originally defined by the embryologist Conrad Waddington, epigenetics is the branch of biology that studied the interactions between genes and their products that bring phenotype into being [109]. Epigenetic information is controlled by genome sequence, environmental exposure, and stochasticity, or random chance. Thus, epigenetics stands at the interface of the genome, development, and environmental exposure [35]. A major change in the epigenetic concept came from the realization that the environment has a profound effect on developmental plasticity, particularly with aging and susceptibility to common disease [110]. The vast majority of human cancers harbor both genetic and epigenetic abnormalities, with fascinating interplay between the two [33]. A key facet of epigenetics is that its modifications can be stably maintained yet adapt to changing developmental or environmental needs [39]. In contrast to the DNA sequence, the epigenome is relatively susceptible to modification by the environment as well as stochastic perturbations over time, adding to phenotypic diversity in the population [111]. A convincing example of intergeneration dietary epigenetic effects was an experiment involving mice with an insertional mutation in the Agouti locus that controls coat color and weight. These phenotypes are regulated by dietary methionine, the essential amino acid precursor for DNA methylation [112]. In humans, exposure to nicotine and other toxins causes substantial epigenetic changes in smokers, affecting genes involved in normal pulmonary function and cancer [113, 114]. A recent randomized trial showed that dietary fat composition affects DNA methylation in adipocytes [115].

Cancer has long been regarded as a genetic disease. Nevertheless, genetic events occur at low frequency and are thus not a particularly efficient means for malignant transformation [116, 117]. Genome-scale genomic and epigenomic analyses have only recently revealed the widespread occurrence of mutations in epigenetic regulators and the breadth of alterations to the epigenome in cancer cells [33]. It is now clear that genetic and epigenetic mechanisms interact with each other to enable the acquisition of the hallmarks of cancer during tumorigenesis [33, 116, 117]. Disruption of a key epigenetic regulator by mutation leads to an altered transcriptome, multiplying the effect of the single genetic alteration [116].

DNMT3A is recurrently mutated in acute myeloid leukemia (AML) and other myeloid malignancies [118, 119]. TET1 and TET3 are rarely mutated in hematological malignancies. By contrast, large-scale whole-exome sequencing studies by many groups have confirmed that TET2 is one of the most frequently mutated genes in chronic myelomonocytic leukemia (~ 50%), acute myeloid leukemia (~ 20%), and myelodysplastic syndromes (~ 20%) [120]. The majority of missense mutations impair the enzymatic activity of TET2, resulting in decreased 5hmC levels and aberrant DNA methylation [121]. The prognostic value of TET2 mutations in cancer patients remains unclear [121]. Since the initial discovery of IDH mutations in cancer in 2008, recurrent somatic mutations in IDH1 and IDH2 have been identified in different malignancies, including gliomas, thyroid carcinomas, cholangiocarcinomas, sarcomas, and AML [122]. The value of IDH mutations is under debate [123]. Mutations in histone variants H3.3 (H3F3A) and H3.1 (HIST1H3B) have been found in pediatric and adult brain tumors with K27 M and G34R or G34 V mutation hot spots [124, 125]. Mutations were also observed in the ATRS and DAXX genes, which encode proteins responsible for loading of the H3.3 variant into the telomere region [124]. The MLL gene, which encodes one of the H3K4 methyltransferases, has more than 50 translocation function partners in different lineages of leukemia. These rearrangements account for 80% of the cases of infant leukemia and 5–10% of adult leukemia cases and are generally associated with poor prognosis [126]. Loss-of-function mutations of MLL3 have been reported in many different types of cancer. MLL2 is mutated at a very high frequency in B cell follicular lymphoma and diffuse large B cell lymphoma [127]. More information about mutations in epigenetic regulator genes is listed in Table 2.

**Table 2:** The interplay of genetics and epigenetics

	Gene	Major role	Aberrant Changes	Tumor type	Ref
Genetics affect epigenetics	DNMT1	DNA methyltransferase	mutation	CRC	[128]
	DNMT3A	DNA methyltransferase	mutation	hematologic malignancies, mostly AML	[118]
	DNMT3B	DNA methyltransferase	mutation, SNPs	ICF syndrome	[129]
	TET1	5′ methylcytosine hydroxylase	chromosome translocation	CRC, CLL	[130]
	TET2	5′ methylcytosine hydroxylase	mutation	myeloid malignancies	[131]
	IDH1/2	isocitrate dehydrogenase	mutation	AML, glioma	[132, 133]
	ALKBH3	DNA dealkylation repair enzyme	mutation	papillary thyroid cancer	[134]
	SETD2	histone lysine methyltransferase	mutation	pleural mesothelioma, lung cancer, leukemia, renal cancers, glioma	[135–138]
	CREBBP	histone acetyltransferase	mutation, amplification	ALL, CRC, GC, lymphoma	[139–142]
	MLL1	H3K4 histone methyltransferase	fusion protein	ALL, AML, B cell lymphoma, prostate cancer, bladder transitional cell carcinoma	[143]
	EP300	histone deacetyltransferase	mutation	myeloproliferative neoplasms, GC, lymphoma, thyroid cancer	[141, 142, 144, 145]
	DOT1L	histone lysine methyltransferase	mutation, amplification	GC, ovarian cancer	[146, 147]
	EZH2	H3K27 Histone methyltransferase	mutation	melanoma, hematologic malignancies, mostly B-cell lymphoma	[148–151]
	NSD1	nuclear receptor binding SET domain protein 1	mutation, frame shift, translocation	CRC, GC, clear cell renal cell carcinomas, head and neck cancers, AML, HCC, malignant peritoneal mesothelioma	[152, 153]
	HDAC10	histone deacetyltransferase	frame-shift deletion	thyroid cancer	[145]
	JMJD1C	H3K4/H3K9 histone demethylase	mutation	clear cell renal cell carcinoma, intracranial germ cell tumors	[154]
	ATRX	member of SWI/SNF family proteins	mutation	adult diffuse astrocytic tumors, glioma, pancreatic neuroendocrine tumors, myxofibrosarcoma, pediatric osteosarcoma	[155–158]
	CHD5	ATPase of NURD	mutation	BC, CRC, neuroblastoma, glioma, lung, ovary, prostate cancers	[159]
	HELLS	helicase involving DNA strand separation	mutation	CRC, GC, NSCLC	[160, 161]
	SMARCB1	BAF subunit	mutation	malignant rhabdoid tumor, epithelioid sarcoma, ovarian small cell carcinoma	[162]
	ARID1A	BAF subunit	mutation, genomic rearrangement	HCC, GC, CRC, BC, endometrial cancer, ovarian clear cell carcinomas, melanoma, bladder, lung and pancreatic cancers	[162]
	SMARCA4	ATPase of BAF	mutation	rhabdoid tumors, epithelioid sarcoma, ovarian small cell carcinoma	[163, 164]
Epigenetics affect genetics	SAT2	satellite 2 repetitive element	hypomethylation	BC, GC, HCC, BC, pancreatic cancer, glioblastoma	[165–169]
	ALU	repetitive element	hypomethylation	BC, CRC, GC, glioma	[170]
	LINE-1	long interspersed nuclear element-1	hypomethylation	CRC, BC, lung cancer, prostate cancer, liver cancer, ovarian cancer, and esophageal cancer	[171]
	MBD4	methyl binding protein	promoter hypermethylation	CRC, ovarian cancer	[172]
	MGMT	O6-meG alkyltransferase	promoter hypermethylation	NSCLC, CRC, GC, gliomas, diffuse large B-cell lymphoma, esophageal and head and neck cancers	[173]
	TDG	thymine DNA glycosylase	promoter hypermethylation	Multiple myeloma	[174]
	NEIL1	removes thymine glycol	promoter hypermethylation	CRC, NSCLC, head and neck cancers	[175–177]
	OGG1	8-oxoguanine DNA glycosylase	promoter hypermethylation	BC, CRC	[175, 178]
	XPC	XPC complex subunit, binds DNA distortions	promoter hypermethylation	bladder cancer	[179]
	XPG	subunit of ERCC5/XPG/Rad2 NER complex	promoter hypermethylation	CRC, ovarian cancer	[180]
	MLH1	MutL homologs, MMR ATPase, forming heterodimer	promoter hypermethylation	ESCC, GC, CRC, NSCLC, ovarian, pancreatic, oral squamous, head and neck cancers, adult T-cell leukemia/lymphoma	[9, 181–186]

In addition to genetic disruption of epigenetic machinery, aberrant epigenetic changes may cause genetic abnormality. Epigenetic silencing of DNA repair genes such as MLH1, MGMT, BRCA1, FANCF, CHFR and SLFN11 can lead to gene mutation and genomic instability in cancer cells [181, 187–189]. Microsatellite instability (MSI) in Lynch syndrome results from germline mutations in mismatch repair genes, mainly MLH1 and MSH2. Approximately 15% of sporadic colorectal cancer patients with MSI were caused by epigenetic silencing of the MLH1 promoter region [190]. MSI caused by epigenetic silencing of MLH1 has also been reported in other types of cancer, including about a quarter of sporadic endometrial cancers [191]. Methylation of MGMT in colorectal cancer is associated with G-to-A mutations in the KRAS gene [192]. Additional epigenetically silenced DNA damage repair genes are listed in Table 2.

## Heterogeneity of cancer epigenetics

Although researchers are still at the very beginning of understanding the full context of tumor heterogeneity, models of tumor evolution, or tumor phylogenies, derived from ITH have improved our understanding of tumorigenesis [2, 24]. A majority of cancer therapies fail to achieve durable responses, which is often attributed to ITH. Importantly, most strategies for cancer therapy still do not assess ITH and miss an opportunity to examine the prognostic value of ITH. ITH has been assessed with somatic mutation and copy-number alteration. The causal relevance of epigenetic changes in cancer has been recognized and the concept of epigenetic silencing being involved in Knudson’s two-hit theory has been accepted [60]. Thus, epigenetic mechanisms play an important role in tumor heterogeneity.

Some studies were not designed a priori for the purpose of analyzing heterogeneity; however, they provided information on epigenetic intratumor heterogeneity (eITH) and linked to clinical outcome. The “field defect” is one example [193]. In many cancers, cells have been shown to acquire pro-tumorigenic mutations that are not able to produce morphological change but predispose cells to subsequent malignant transformation [194]. These cells can expand, creating patches of mucosa that have an increased risk of developing into cancer. This process has been described as “field cancerization” or “field defect” [11, 195]. Epigenetic abnormalities may serve as a marker of a “field defect”, such as MGMT, p16 and RASSF1A promoter region methylation in normal-appearing mucosa of colorectal cancer patients [193, 196].

eITH can be examined at the level of histone modifications, chromatin conformation, or DNA methylation. Nevertheless, epigenetic heterogeneity remains poorly explored. To date, DNA methylation has been the major measurement due to the quantitative nature of DNA methylation assays and the relative ease of obtaining sufficient genomic DNA compared to chromatin. Using human cutaneous melanoma as a model, Sigalotti et al. found that the expression of cancer/testis antigens (CTA) was highly heterogeneous in different clones, which were generated from a melanoma lesion metastasized to the lymph-node. In addition, the clonal heterogeneity of CTA expression was negatively correlated with promoter region hypermethylation [197]. By analyzing the promoter region methylation of five genes (RASSF1A, p16, DAPK, MGMT, and Rb) in 34 tumors (15 melanoma primaries, 19 metastases), Rastetter et al. found that 70% of the cases exhibited heterogeneous methylation patterns [198]. In another study, in nine MSI-positive primary endometrial cancers that lacked MLH1 expression based on immunohistochemical evaluation, eight of nine tumors were methylated in the promoter region. Among these, four tumors were homogeneously methylated and four cases were heterogeneously methylated [199]. Genomic sequencing of 28 chronic lymphocytic leukemia (CLL) patients where samples were taken at two or more time points, Okes et al. found that CLL cases that showed high levels of genetic heterogeneity also showed widespread methylation changes over time [200]. While, Pan et al. found that intratumor methylation heterogeneity does not clearly correlate with genetic clonal heterogeneity in diffuse large B-cell lymphomas according to enhanced reduced representation bisulfite sequencing [201]. Aryee et al. reported that cancer-related genes are heterogeneously hypermethylated across individuals in prostate cancer patients, while the methylation status is generally consistent across metastases within individuals. However, some regions showed intraindividual metastatic tumor heterogeneity in promoter methylation, and such methylation alterations were generally not correlated with gene expression. This is likely due to the complexity of tumor components and/or one allele methylation [202]. By analyzing 40 tissue samples from seven lung adenocarcinoma patients (including normal, tumor segments and lymph node metastases), a recent study found that methylation profiles within tumors from same individual were not more similar to each other than to those from others [203].

Using Illumina Human Methylation 450 k BeadChip arrays, Brocks et al. analyzed DNA methylation and copy number alterations from multiple topographically distinct tumor sites in 5 patients, including primary tumor sites, premalignant lesions, lymph node metastases and matched normal prostate epithelium. They demonstrated that both DNA methylation and copy-number heterogeneity consistently reflect the life history of the tumors [204]. In this study, specimens of the same patient were generally more similar to each other than those from different individuals, consistent with the previously described interindividual heterogeneity of prostate cancer metastases [201, 205, 206]. Further study suggested that intratumor heterogeneous DNA methylation presents in multiple subclonal cell populations. In addition, metastases always carried unique alterations not found in the primary tumor bulk, and metastases-specific aberrant methylation events frequently co-localized with genes involved in metastases-associated processes. The degree of intratumoral DNA methylation variability strongly depends on the genetic and epigenetic context of a locus [204]. By performing a multitude of analyses of the DNA methylation data in tumors and normal samples of 21 cancer types in TCGA, Liu et al. revealed that the variability of the DNA methylomes are highly enriched in the gene promoters of the DNA-binding proteins, especially the transcription factors (TFS) [207]. Combining single-cell profiling of expression and DNA methylation, Linker et al. found DNA methylation is locus-specific, and methylation heterogeneity across cell is associated with splicing variability [208]. Quek et al. analyzed methylation profiles of 48 spatially separated tumor regions from 11 localized lung adenocarcinomas and their matched normal lung tissues using the Illumina Infinium Human Methylation k450 BeadChip array. All tumor samples had at least 40% viable cancer cells, and only approximately 25% of all differentially methylated probes were clonal events shared by all regions of individual tumors, and a higher extent of DNA methylation ITH was associated with larger tumor size [209]. Martinez-Cardus et al. found that intratumor DNA methylation differences are more extensive than genetic diversity in primary colorectal cancer. They also revealed that those locoregional colorectal cancer tumors more homogeneous at the epigenetic level show poor clinical outcomes [210]. To determine the prevalence and character of epigenetic tumor heterogeneity in time and space, DNA methylation sequencing was performed on a large cohort of IDH wildtype glioblastoma patients (*n* = 112) with mathched samples from primary and recurring tumors (between 2 and 4 time points per patient), including multiple subregion samples for a set of these tumors. By comparing DNA methylation levels of 5-kilobase tiling regions between primary and recurring tumors, Klughammer et al. observed wide-spread epigenetic heterogeneity at individual loci [211]. An example is that the MGMT promoter was unmethylated in the majority of samples, and patients with a methylated MGMT promoter in their recurring tumors had significantly better progression-free survival (PFS) and overall survival (OS) compared to patients with unmethylated MGMT promoters. A demethylation of Wnt signaling gene promoters was associated with worse prognosis. Extensive heterogeneity existed between patients, but not strong trend between primary and recurring tumors. Increased epigenomic heterogeneity was associated with worse prognosis. Authors also found that DNA methylation data could predict various types of immune cells in the primary and recurring tumors [211]. Developing a novel single-cell technology, Pi-ATAC, which simultaneously measures protein epitopes and active DNA regulatory elements of the same individual cell, Chen et al. found epigenetic variability of tumor cells is linked to the hypoxic tumor microenvironment [212]. By genome-wide methylotyping analysis, Tanas et al. divided breast cancer into six breast cancer methylotypes, and found that the majority of CpG islands appeared to be more densely hypermethylated in breast cancer cell lines than in primary tumors [213]. Using an epigenome-wide sequencing approach, Grasse et al. observed that aberrantly methylated regions in the PDX tumors were reflected in the corresponding primary NSCLC tumors, albeit the levels of differential methylation of the PDX samples were much higher compared to the levels within the primary tumors [214]. Mutations in epigenetic modifier genes, such as SETD2 and DNMT3A, are strongest determinants of ITH amongst a panel of 17 distinct cellular pathways [215]. Epigenetic regulators such as histone modifying enzymes are critical for the establishment of cell-type-specific gene expression patterns, thus, they are also likely to play a role in modulating cell-to-cell variability in transcription. The distinct epigenetic state of the cells could determine cellular response to treatment [216]. Lysine demethylase 5 (KDM5) was found to be a regulator of cellular transcriptomic heterogeneity in ER^+^ luminal breast cancer, and inhibiting KDM5 activity could decrease resistance to cancer therapies [217]. Pastore et al. suggested that intratumoral epigenetic diversity may permit leukemic cells to stochastically activate alternate gene regulatory programs, facilitating the emergence of novel cell sates, ultimately fostering CLL’s ability to efficiently explore the fitness landscape for superior evolutionary trajectories during tumorigenesis and in response to therapy [218].

Genome-wide sequencing of three cases of primary melanoma and matched metastatic cell lines derived from the same patients showed global hypomethylation in metastatic melanoma cell lines compared to the matched primary melanoma cell lines [219]. A recent study found that the activation-induced cytidine deaminase (AICDA) is a key driving force in generating cytosine methylation heterogeneity in germinal center B cells and GC-derived lymphomas. AICDA-linked epigenetic heterogeneity is predominantly associated with relative loss of cytosine methylation. AICDA-induced epigenetic heterogeneity increases plasticity, permitting cancer cells a greater degree of population diversity and enhancing the adaptive capacity of the overall tumor. AICDA overexpression in mice was associated with both increased inter-tumor and intra-tumor methylation heterogeneity [220].

## The strategies of “epigenetic precision medicine” based on cancer heterogeneity implications

In contrast to the “one-size-fits-all-approach”, the ultimate aim of precision medicine is to enable clinicians to accurately and efficiently identify the most effective preventive or therapeutic intervention for a specific patient. A variety of high-throughput methods for characterizing cancer biomarkers (proteomics, genomics, epigenetic, transcriptomics), coupled with significant advances in computational tools, may improve understanding precision medicine in cancer [221]. Epigenetic switches play an important role in cancer development, and epigenetic switches are reversible. Thus, aberrant epigenetic changes may serve as early detection, prognostic and chemo-sensitive markers in cancer. They may also become preventative and therapeutic targets in cancer [26, 35]. One example is that an “epigenetic field defect” is formed during chronic inflammation-associated carcinogenesis, and aberrant DNA methylation is induced by chronic inflammation. DNA methylation was induced in colonic epithelia cells as early as 8 weeks after dextran sulfate sodium (DSS) treatment when no macroscopic tumors appeared, and the methylation level gradually increased until macroscopic tumors developed. Our previous study and others suggest that “epigenetic field defect” may serve as an early detection marker in cancer [201, 222]. Elucidation of the specific epigenetic marker that underlies the epigenetic heterogeneity could enable specific chemo-preventative agents to be designed to target these early changes prior to the development of any precancerous lesions. Several studies noted that the width of the surgical margin is directly associated with the risk of local recurrence (or development of invasive cancer) following breast conserving surgery for ductal carcinoma in situ (DCIS) [223]. These findings are consistent with the idea that aberrant epigenetic changes may exist in histologically normal appearance epithelia cells around the lesions.

Heterogeneity of the tumor microenvironment may also result in diversity of tumor cell phenotypes, which decreases the sensitivity of the tumor to therapy. For instance, under conditions of hypoxia, tumor cells are more aggressive and their response to treatment is worse than in normally oxygenated regions [224]. Aryee et al. found that there is a considerable amount of interindividual tumor heterogeneity at both the genetic and epigenetic levels in prostate cancer [202]. This interindividual heterogeneity challenges “one-size-fits-all” approaches for cancer management and implies the need for specific treatment for different molecular lesions. The finding of metastases-specific aberrant methylation and identification of high levels of epigenetic heterogeneity at androgen-receptor-bound enhancer domains adds information about regulatory activity at important cis-regulatory elements and assists in making decisions for precision medicine strategies in prostate cancer [204]. Overexpression of AICDA, a driver of epigenetic heterogeneity, is associated with a more aggressive disease phenotype and decreased survival in BCL2-driven lymphoma [220]. Clonal evolution of multiple myeloma cells and heterogeneity of the bone marrow microenvironment results in a rapid acquisition of chemotherapy resistance.

The central role of epigenetics in regulating many of the hallmarks of cancer has garnered the interest and focus of scientists, clinicians, and the pharmaceutical industry with the aim of manipulating and resetting the cancer epigenome. In the past few years, plenty of small molecules have been developed to target specially epigenetic writers, readers, and erasers [225]. The DNA demethylating agents 5-azacytidine and 5-aza-2’deoxycytidine (decitabine) are inhibitors of DNMT1 and DNMT3B. Decitabine has been approved by US FDA for myelodysplasia and AML treatment, and 5-azacytidine has also been approved for myelodysplasia therapy. Guadecitabine (SGI-110) is a second-generation demethylating agent, which is more stable in aqueous solution, and more demethylating agents are being testing in solid tumors. Trichostatin A (TSA) is the first natural product discovered to inhibit HDACs. There are currently at least 20 HDAC inhibitors in clinical testing. Vorinostat (also known as suberoylanilide hydroxamic acid [SAHA]) and romidepsin (also known as depsipeptide or FK228) were approved by the FDA for treatment of cutaneous T-cell lymphoma. Enhancer of zeste homologue 2 (EZH2) is the catalytic core subunit of the polycomb repressive complex 2 (PRC2). It is responsible for catalyzing trimethylation of histone H3 at lysine 27, which serves as a docking site for DNA methyltransferases and HDAC. As the C-terminal SET domain of EZH2 exhibit methyltransferase activity, specific inhibitor has been designed by targeting the conserved SET domain. A batch of SET domain inhibitors is being selected to minimize the off-target effects. GSK126 and EPZ-6438 are being tested in phase I trial in solid tumors. Disruptor of telomeric silencing-1-like (DOT1L) is a methyltransferase responsible for catalyzing methylation of H3K79. MLL-fusion proteins gain the ability to recruit DOT1L to MLL target genes, leading to aberrant expression of these genes by methylating H3K79. EPZ00477 and EPZ-5676 are inhibitors of the human DOT1L. The phase I clinical trial of EPZ-5676 has been completed in MLL-rearranged leukemia [226]. The methylation status of histone lysine is controlled by KDMs and their counterparts of lysine methyltransferases (KMTs). Lysine specific histone demethylase-1 (LSD1, also known as KDM1) catalyzes the demethylation of mono- and dimethylated lysines, but not tri-methylated lysines from H3K4 and H3K9. LSD1 was found to be highly expressed in neuroblastoma, prostate, estrogen-negative breast, bladder and colorectal cancers. GSK2879552 and ORY-1001 are specific inhibitors of LSD1. They are currently in clinical trials for small cell lung carcinoma and relapsed or refractory AML, respectively. Based on the JmjC domain sequence homology and their demethylase activities, JmjC-KDMs have been categorized into seven KDM subfamilies (KDM2–8). KDM5 members are capable of removing H3K4me3 activating mark from histones to make them potential players in the downregulation of tumor suppressors. Inhibition of KDM5 demethylase activity reduces the number of surviving cells after lethal drug exposures in a number of cell culture models, what makes this enzyme family a promising target for novel cancer treatment [227]. The bromodomains (BRDs) may contribute to highly specific histone acetylation by tethering transcriptional HATs to specific chromosomal sites, or to the activity of multiprotein complexes in chromatin remodeling. The bromodomain and extra-terminal motif (BET) proteins act as scaffolds for the recruitment of transcription factors and chromatin organizers required in transcription initiation and elongation. Extensive studies have explored small-molecule inhibitors of BET family proteins for cancer therapy. I-BET762 is being tested in early phase clinical trials. More clinical trials are performing for BET family inhibitors, including RVX-208, I-BET 762, OTX 015, CPI-0610 and TEN-010. There are more epigenome-based targeting therapeutics, but they are beyond the scope of this review [26, 225].

Epigenetic heterogeneity is far more dynamic than genetic heterogeneity, and it is likely that transcriptional plasticity driven by epigenetic regulators responding to environmental and therapeutic pressures underpins the failure of many cancer drugs to induce durable disease remission in patients [225]. Several classes of epigenetic regulators have been implicated in drug resistance and intratumoral heterogeneity [228]. Epigenetic therapy may reverse drug resistance in heterogeneous multiple myeloma [229]. Combination of epigenetic therapy and chemotherapy improved the efficacy in refractory advanced non-small cell lung cancer [230]. Ideal treatment regimens would target all the different subpopulations of cancer cells present at the time of treatment, thus avoiding resistance and delaying relapse [228]. By detecting genetic and epigenetic heterogeneity and analyzing compensatory signaling in cancer, we may develop novel “synthetic lethality” strategies. As cancer epigenetic heterogeneity is in its infancy, little can be generalized from epigenetic heterogeneous therapy.

## Conclusions & future perspectives

ITH may reflect the evolutionary history of tumors, and genetic or epigenetic marks can also reflect the potential of the tumor to respond to an environmental or therapeutic pressure. Understanding ITH may guide new therapeutic strategies [21]. The current trend of epigenetic therapy is to use epigenetic drugs to reverse and/or delay future resistance to cancer therapies. As epigenetic abnormalities are apparent early in cancer risk and premalignant states, we may be able to develop strategies for cancer prevention. One of the major issues in elucidating the “road map” of human development and disease epigenomes is technique limitation. However, a new generation of sequencing instrument is in development. Nanopore sequencing is a third generation sequencing technology that assesses single molecules of unmodified DNA by sensing alterations in electrical current that occur as different bases pass through a nanopore. Oxford Naonopore Technologies has recently released the first commercially-available sequencer based on this technology. This technology accepts samples as small as 10 pg and does not require PCR amplification prior to analysis. Nanopores are also capable of distinguishing between cytosine, 5mC, and 5hmC [231]. Ideally, in vivo and in vitro tumor models that recapitulate the nature, dynamics, and heterogeneity of successive tumorigenic epigenetic alternations are needed [232]. Epigenetics may lead us at last to an era of comprehensive medical understanding, unlocking the relationships among the patient’s genome, environment, prenatal exposure, and disease risk in time for us to prevent diseases.

Many questions about epigenetic heterogeneity in cancer remain to be answered. In nearly every study to date, the proportion of a tumor that is assayed is quite small relative to the full tumor mass in the patient. ITH may explain the difficulties encountered in the validation of oncology biomarkers and prediction of therapeutic resistance owing to sampling bias [24]. Current measures of eITH significantly underestimate the levels of ITH, and signals from bulk tumor samples are dominated by major subclones, rendering rare subpopulations undetectable [23]. eITH may reflect a mix of subclones with distinct genomic and epigenomic features. In addition, epigenome variability comes from a variety of other cells present in tumor tissues, including nontumor stromal and immune cells. A plethora of newly identified mutations in epigenetic regulators remain largely uncharacterized. It is necessary to identify them to be epigenetic drivers/passengers by functional experiments [23]. The tumor microenvironment may represent as much as 90% of some tumor samples and contribute proportionally to the RNA pool, which affects measures of heterogeneity and resulting transcriptional profiles. Thus, both the tumor and its microenvironment, including tumor-infiltrating leukocytes, should ideally be assayed. Epigenetic modifications are dynamic and responsive to environmental pressures, and they may reflect the potential of the tumor to respond to an environmental or therapeutic pressure [21]. Complete understanding of genetic and epigenetic heterogeneity may assist in designing combinations of targeted therapies based on molecular information extracted from individual tumors. Ideally, we could always target druggable trunk mutations/aberrant epigenetic changes, and then add drugs to target emerging subclones.

## Data Availability

The material supporting the conclusion of this review has been included within the article.

## Abbreviations

5mC: 5-methylcyctosine
AICDA: Activation-induced cytidine deaminase
ALL: Acute lymphoblastic leukemia
AML: Acute myeloid leukemia
BC: Breast cancer
BER: Base excision repair
BET: The bromodomain and extra-terminal motif
BRDs: Bromodomains
CAFs: Cancer-associated fibroblast
CLL: Chronic lymphoblastic leukemia
CRC: Colorectal cancer
DCIS: Ductal carcinoma in situ
DNMTs: DNA methyltransferases
DOT1L: Disruptor of telomeric silencing-1-like
DSS: Dextran sulfate sodium
ESCC: Esophageal squamous cell carcinoma
EZH2: Enhancer of zeste homologue 2
FA: Fanconi anemia
GC: Gastric cancer
HATs: Histone acetyltransferases
HCC: Hepatocellular carcinoma
HDACs: Histone deacetylases
HDMs: Histone demethylases
HMTs: Histone methytransferases
HR: Homologous recombination
ITH: Intratumor heterogeneity
KDMs: Lysine demethylases
KMTs: Lysine methyltransferases
LSD1: Lysine specific histone demethylase-1
MMR: Mismatch repair
MSI: Microsatellite instability
NER: Nucleotide excision repair
NHEJ: Non-homologous end-joining
NSCLC: Non-small cell lung cancer
OS: Overall survival
PFS: Progression-free survival;
PRC2: Polycomb repressive complex 2
TDG: Thymine-DNA glycosylase
TET: Ten-eleven translocation
TFS: Transcription factors
TSA: Trichostatin A
TSGs: Tumor-suppressor genes
TSS: Transcription start sites
